# Direct Analysis of Bio-Molecules in Solid Materials Using Electrospray Ionisation Mass Spectrometry Coupled with Laser Ablation and a Liquid Sampling Technique

**DOI:** 10.5702/massspectrometry.A0121

**Published:** 2023-05-10

**Authors:** Takafumi Hirata, Menghao Yang, Hui Hsin Khoo

**Affiliations:** 1 Geochemical Research Center, The University of Tokyo, 7–3–1 Hongo, Bunkyo-ku, Tokyo 113–0033, Japan

**Keywords:** laser ablation in liquid, direct solid sampling, ESI-mass spectrometry, high recovery extraction, analysis of organic compounds

## Abstract

*In situ* and rapid analysis of organic compounds using a combination of a newly-developed laser ablation in liquid (LAL) sampling technique combined with electrospray ionisation mass spectrometry (ESI-MS) is reported. The LAL is a technique that allows laser ablation to be conducted in a liquid medium containing organic compounds that were effectively extracted from solid materials into the liquid medium. Three organic compounds (valine, caffeine, and benzyl butyl phthalate (BBP)) were subjected to analysis. The LAL sampling was conducted in the fast-laser scanning mode using Galvanometric optics, and the total ablation time required for the sampling from a 1 mm^2^ area was about 3 s, thus providing rapid sampling. The resulting sample solution was directly introduced into the ESI-MS system, without the need for any chromatographic separation. To evaluate the analytical capability of the LAL technique coupled with ESI-MS, both the overall transmission efficiencies of analytes from solid materials to the ion detector, and the repeatabilities of the measurements were rigorously tested. This involved the use of synthetic, in-house prepared standard materials containing the analytes. The overall ion yields were about 1.1×10^−3^% for valine, 8.7×10^−3^% for caffeine, and 6.7×10^−4^% for BBP. By comparing the ion yields obtained by the injection of an analyte solution and a standard solution through the mass spectrometer, the recoveries through the LAL sampling were approximately 31% for valine, 45% for caffeine, and 37% for BBP. In addition, the analytical repeatabilities for all analytes were better than 6%. The analytical repeatabilities were mainly affected by either the heterogeneity of the in-house standard materials or changes in the plasma temperature by coexisting, laser-induced sample particles. It should be noted that not only water-soluble compounds (caffeine and valine), but also non-soluble compound (BBP) could be measured by the LAL-ESI-MS, which is one of the great advantages over the conventional liquid extraction surface analysis technique. The data obtained here clearly demonstrate that the LAL-ESI-MS has the potential for being a fast and user-friendly analytical technique for the *in-situ* detection for both the water-soluble and water-insoluble molecules.

## INTRODUCTION

### *In situ* sampling

Mass spectrometry is now one of the principal analytical tools for the *in situ* detection of various organic compounds, from small molecules to large biomolecules. Various combinations of sampling probes and ion sources have been applied for the measurements of these molecules. Among them, mass spectrometry coupled with matrix-assisted laser desorption/ionisation mass spectrometry (MALDI/MS) is the most widely applied approach for the *in situ* sampling and imaging analysis of molecules. In the MALDI technique, the target molecules are extracted into the matrix components, and the molecules are then ionised through a protonation process, resulting in mainly protonated ions fragmentation being a minor contributor.^1–5)^ Despite the obvious success in the soft ionisation of molecules by the MALDI technique, the technique requires technical skills for sample preparation, which includes matrix selection and application. An improper choice or coating of the matrix materials can cause a low signal-to-background ratio (SBR) and poor analytical repeatability.

**Figure 1:**
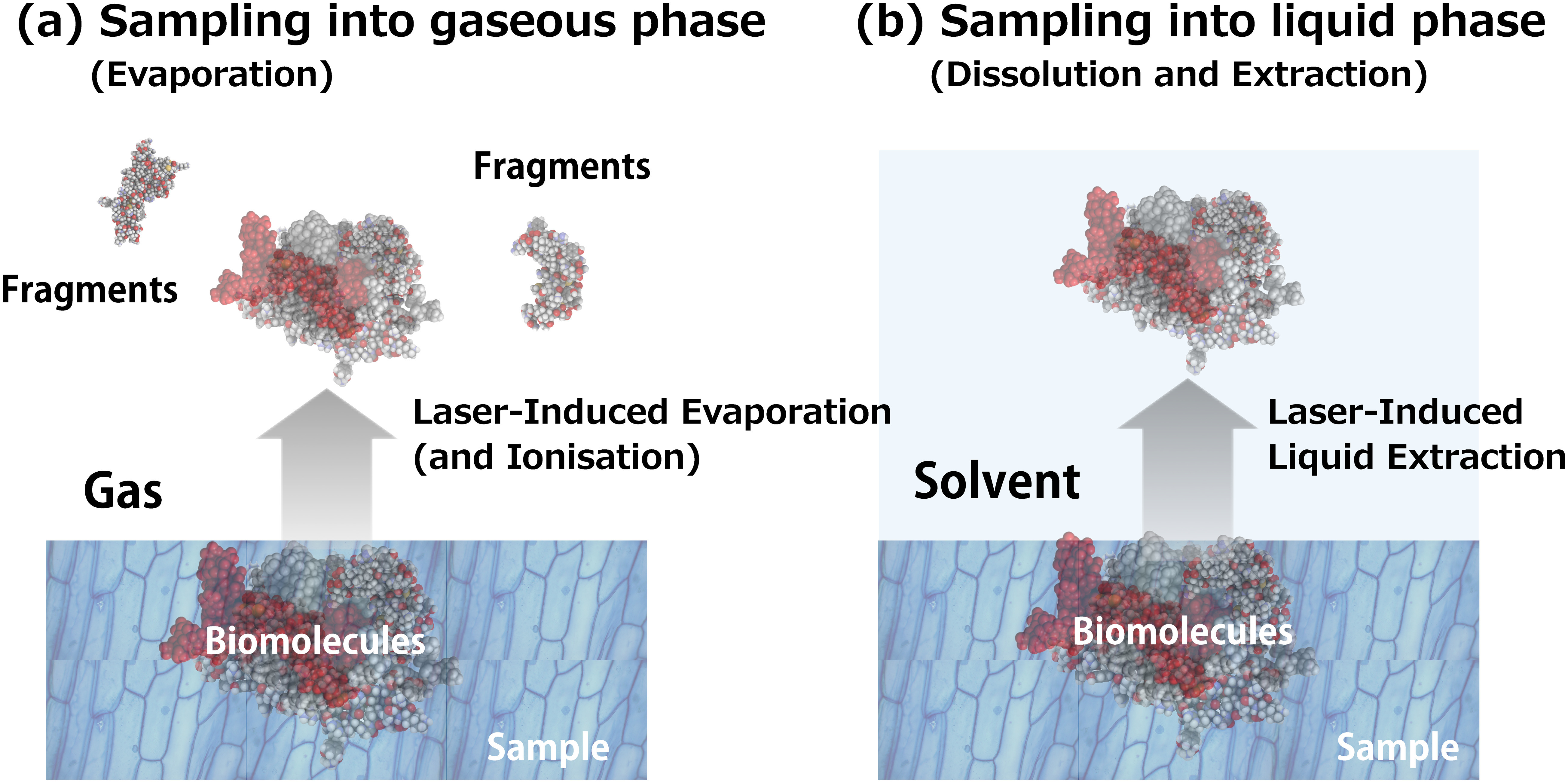
Fig. 1. Comparison of the sampling and extraction of molecules into the (a) gas phase and (b) liquid phase. Less energy is required for dissolution, suppressing fragmentation of molecules.

Another choice for the *in situ* analysis of organic compounds is electrospray ionisation-mass spectrometry coupled with a laser ablation sampling technique (LAESI-MS).^6)^ The sample surface is irradiated with the laser, and the induced sample particles or vapour containing the analyte and ionisation takes place through the collision of sample molecules and the electrospray plume formed by the ESI. Since laser ablation is conducted under the atmospheric pressure, the technique is inherently suitable for *in situ* sampling from tissues and living cells. Using LAESI-MS, mass spectrometric studies of biomolecules can be made with minimal sample preparation, thus eliminating the need for the time-consuming surface coating of conducting materials or matrix components. Despite the several unique features of this technique, there are several drawbacks such as (1) low ionisation efficiency of the analytes, (2) poor repeatability of the measurements, and (3) potential fragmentation through evaporation. Among these problems, one of the major problem associated with MALDI and ESI is that ionisation is carried out in the gas phase, so the target molecules must be evaporated prior to ionisation.^4–6)^ Since the energy required for evaporation (∆*G*_evaporation_) would be greater than that for dissolution (∆*G*_dissolution_), a higher energy load onto the sample can cause the fragmentation of molecules. This suggests that dissolution would be a more effective extraction or sampling technique for analytes with a smaller contribution of fragmentation (Fig. 1). This was described in the recently-introduced liquid extraction surface analysis (LESA).^7)^

The LESA technique is a discrete, ambient sampling technique for the analysis of biomolecules from a sample surface. Organic compounds are directly extracted into small droplets placed on sample surface, and the droplets are then injected into ESI ion source.^8)^ The LESA technique allows the direct analysis of folded proteins and protein complexes from biological substrates *via* the use of native-like extraction/ionisation. With denaturing LESA solvents, the highest mass protein detected in tissue to date was greater than 70 kDa.^9)^ The distinct advantage of LESA is the ability to simultaneously extract multiple types of analytes from a single location with little to no need for sample preparation under atmospheric pressure. LESA has been used to characterise proteins in a wide array of samples, including dried blood spots, tissue sections, even living bacterial colonies.^10,11)^ Despite these analytical advantages, there are two major drawbacks to this technique. The first is that the spatial resolution achieved by the LESA technique (*ca.* 1 mm) is significantly lower than those achieved by various *in situ* analytical methods such as MALDI/MS (*ca.* 5–30 μm), LAESI-MS (*ca.* 30 μm), direct liquid extraction and ionisation technique including liquid micro junction surface sampling probe/electrospray ionisation mass spectrometry (100 μm) secondary ion mass spectrometry (*ca.* 1–10 μm), fast atom bombardment-mass spectrometry (*ca.* 50 μm), the laser-induced desorption ionisation mass spectrometry (*ca.* 30 μm).^5,6,12–15)^ To expand the analytical capabilities of the LESA technique, further improvements in spatial resolution are required. Another problem associated with the LESA technique is that the target molecules should be soluble to the solvents being used, and, as a result, the sampling efficiency for the insoluble compounds can become low. This suggests that soluble components can be preferentially sampled, resulting in the technique being incapable of simultaneous multicomponent analysis. To overcome these problems, we developed new sampling technique based on laser ablation in a liquid.

### Laser ablation in a liquid (LAL) technique

**Figure 2:**
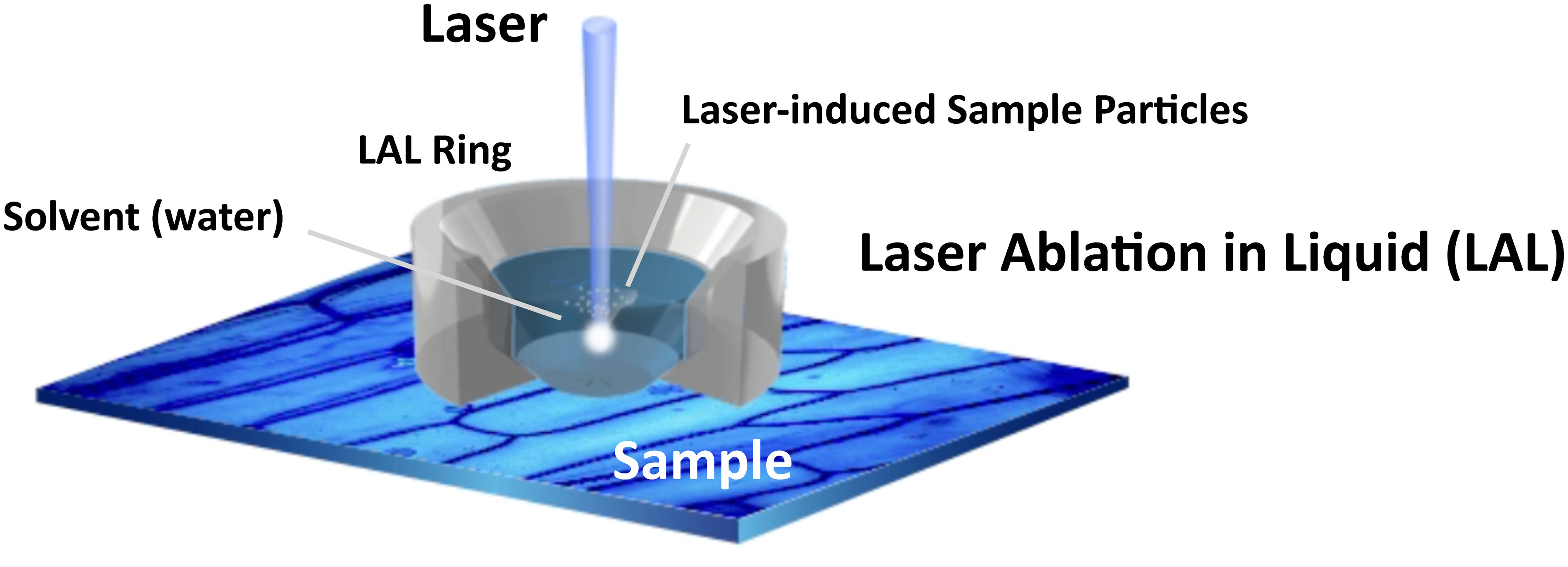
Fig. 2. Schematic diagram for laser ablation in liquid (LAL) technique.

Laser ablation in a liquid (LAL) is widely used for the micro fabrication or production of nanoparticles of various materials (Fig. 2).^16,17)^ In this study, the LAL technique was used as a sampling technique for organic compounds from solid materials. In the technique, the small ring (ferrule for the Swagelok connector made of polytetrafluoroethylene (PTFE)) is placed on the targeted area of the solid material. After filling the ring with 100 μL of deionised water, laser ablation is conducted onto the solid materials through the liquid. The laser-induced sample particles are then directly trapped and collected in water with a level of efficiency.^18,19)^

Laser ablation is directly conducted in a liquid medium. The laser-induced sample particles are trapped/collected in the liquid, reducing sample loss.

As the name indicates, the sampling mechanism, including sample heating and aerosol production of the solid materials through the LAL technique is basically consistent with that of LA. This was also confirmed by time-resolved shadow images.^20)^ However, because of the difference in physical and chemical properties between the gas phase and the liquid phase, the mechanisms of laser ablation in these two phases are, to some degree, different. Within a few picoseconds after the arrival of the laser pulse, laser ablation in the gas or liquid phase is very similar. The important differences, however, are observed subsequently. Unlike in the gas phase, in the liquid phase, the plasma plume is strongly enclosed in the irradiation region by the liquid, and hence, the cooling rate at the interface between the enclosed plasma plume and the target decreases so that the enclosed plasma plume can provide heat energy for the target below. The production of nanoparticles of the solid material and instantaneous heating can result in the release of organic compounds into the solvent. The use of a laser enables the LAL technique to achieve high spatial resolution for sampling. With the LAL technique, sampling can be made with a pit size of 10 μm. Moreover, instantaneous heating of the laser induced sample particles can promote the dissolution of insoluble compounds in liquid phase. In this study, the LAL technique was applied as a new sampling technique for mass spectrometric analysis using ESI. To test the analytical capability of the LAL technique for molecular analysis, sampling recovery and analysis repeatability for three organic compounds (valine, caffeine, and benzyl butyl phthalate (BBP)) that were present in solid materials were evaluated.

## EXPERIMENTAL

### Choice of analytes

In this study, valine, caffeine, and BBP were used as representative model compounds. Valine is one of the essential amino acids for humans. Valine, like other branched-chain amino acids, is associated with weight loss and decreased insulin resistance,^21)^ and therefore monitoring the levels of valine in blood is important for the detection of diabetes. Caffeine is a central nervous stimulant that can temporarily eliminate drowsiness and restore energy. Caffeine is also the most commonly used psychotropic drug in the world.^22)^ BBP is one of the phthalate esters, and is widely used as a plasticiser for polyvinyl chloride. Animal experiments have shown that BBP can increase the incidence rate of pancreatic tumors in mice and is toxic to aquatic organisms,^23,24)^ and hence, the rapid and sensitive detection of the BBP from polymers is highly desired. The molar masses are 117.15 g mol^−1^ for valine, 194.19 g mol^−1^ for caffeine, and 312.365 g mol^−1^ for BBP. Both the valine and caffeine are soluble in water, whereas the BBP is nearly insoluble in water (solubility being <0.1 mg mL^−1^ at room temperature). Because BBP is only sparingly soluble, the sensitive detection of BBP is difficult using the conventional LESA technique.

### Chemicals and reagents

L-Valine (Fujifilm Wako Pure Chemical Corporation, Tokyo, Japan), caffeine, anhydrous (Fujifilm Wako Pure Chemical Corporation, Tokyo, Japan), BBP (Sigma Aldrich, St. Louis, MO, USA), titanium(IV) oxide (Anatase Form, Kanto Chemical Co., Inc., Tokyo, Japan), calcium carbonate (Kanto Chemical Co., Inc., Tokyo, Japan), ethanol (Fujifilm Wako Pure Chemical Corporation, Tokyo, Japan), methanol (Fujifilm Wako Pure Chemical Corporation, Tokyo, Japan), formic acid (Fujifilm Wako Pure Chemical Corporation, Tokyo, Japan), photocurable resin (Acryl One #2100N, Maruto Instrument Co., Ltd., Tokyo, Japan) were used for preparation of the solid standard materials.

### Preparation of standard materials

For ESI, the choice of solvent is important in terms of improving the efficiency of ion formation, and therefore, a mixture of water and methanol in a volume ratio of 1 : 1 was used as a standard solution. In addition, 0.1% formic acid was added to the solvent to reduce the initial droplet size. Prior to the analysis, operational conditions for both the ESI and mass spectrometer were optimised by maximising the signal intensities for valine, caffeine, and BBP through the introduction of standard solutions. In this study, three separated standard solutions containing 100 ng mL^−1^ of valine, caffeine, and BBP dissolved in mixture of 50% MeOH and 0.1% HCOOH were prepared.

### Preparation of solid standard materials

The main objective of this study was to demonstrate the analytical capability of LAL-ESI-MS. To evaluate both the transmission efficiencies of analytes and analytical repeatability, solid standard materials containing known concentrations of analytes are needed. Hence, we prepared samples of analytes in a polymer resin. For the LAL technique, coloured materials are more favorable than clear, noncoloured materials since they allow the laser beam to be more easily focused on the sample surface. To accomplish this, CaCO_3_ and TiO_2_ were added to the resin. These substances act as pigments and the resulting resins are opaque white.^25)^ Caffeine and valine are solids, and BBP is a liquid at room temperature, and thus, two different sample preparation methods were employed.

For valine and caffeine, each analyte was mixed with CaCO_3_ and TiO_2_ in a ratio of 1 : 16 : 1. After the addition of a small volume of ethanol, the resulting powder was ground and mixed with a pestle and mortar. The resulting mixed powder was then dried for 24 h at room temperature. About 0.5 g of the mixed powders were then mixed with 4.5 mL of resin, and then placed into a mold with a size of 5×5×3 mm. Each individual resin sample was then polymerised for 2 h under a UV lamp. The resulting resin blocks were milky white, and the resulting concentrations for valine and caffeine in solid materials were estimated to be around 5.5 mg mL^−1^.

For BBP, the standard solid material was prepared with a slightly different approach. Prior to the addition of BBP, a mixed powder CaCO_3_ and TiO_2_ in a ratio of 16 : 1 was prepared. After mixing using a mortar, about 0.025 g BBP was added to 0.5 g of the mixture of CaCO_3_ and TiO_2_. About 0.5 g of the mixed powders were then mixed with 4.5 mL of resin, which was then polymerised in the same manner as was used for the solid standards for valine and caffeine. The concentrations for BBP in solid materials was about 10 mg mL^−1^.

### LAL conditions

Size distribution of the laser induced solid particles is a function of wavelength and the fluence of the laser, and laser ablation with a high fluence can cause the production of large sized particles,^18,26)^ resulting in a lower extraction efficiency of the target molecules into the solvent. Laser ablation with a lower fluence, however, can result in the sampling volume becoming low, resulting in lower analytical sensitivities for the analyte. To compensate for this, laser ablation was conducted with a medium fluence (6 J cm^−2^) and a high-repetition rate (40 kHz). The total sampling area was a 1 mm×1 mm (square-shaped) with 10 μm depth. To minimise sampling time, laser ablation was conducted with a high-scan rate (10 mm s^−1^) using a Galvanometric scanner.^27,28)^

For the LAL technique, the choice of solvent is very important in terms of improving the extraction efficiency of the target molecules from the laser-induced sample particles. Amendola reported that the particle size of the generated nanoparticles can change when different solvents are used during LAL.^29)^ When silver and gold were ablated either by a laser in either ethanol, acetonitrile or dimethylformamide, the resulting size distributions did not vary significantly, and the average size of the produced nanoparticles was smaller than that found in particles produced by LAL using water. Since organic solvents may react with the extracted substances,^30)^ the resulting difference in the size of particles can reflect the interaction or reaction of the laser-induced particles and solvents. To avoid the potential decomposition of the target molecules, deionised water was used as the solvent for the LAL sampling. After the LAL sampling, 100 μL of mixture of 1 : 1 MeOH : H_2_O and 0.1% HCOOH was added to improve the ionisation efficiency in the ESI.

### Sample preparations for ESI

In ESI, the selection of the solvent is particularly important for achieving higher ion yields of analytes. Typical solvents for ESI can vary from polar and protic, to nonpolar and aprotic, but high volatility solvents (*e.g.*, methanol, acetonitrile, tetrahydrofuran, dichloromethane, trichloromethan, isopropanol, and mixtures of these) are usually required. To reduce the initial size of the droplets, low viscosity solvents and volatile acids or bases such as formic acid (HCOOH) are typically added to the sample solution. These compounds can also act as proton sources for protonation. In this study, the collected sample solutions were diluted with a 1 mL mixture of 50 v/v% MeOH and 0.1 v/v% HCOOH prior to the measurements using the ESI-MS.

### Mass spectrometer

The ESI-mass spectrometer used in this study was the QTRAP® 5500 system (AB SCIEX, Toronto, Canada). The data acquisition was conducted in the multiple reaction monitoring (MRM) mode, which improved the SBR for the analytes through collision induced dissociation. One of the great advantages of using the MRM is that multiple compounds can be simultaneously monitored.^11,31)^ Instrumental settings such as temperature at the capillary and collision energy in the Q2 (collision cell) were carefully optimised through monitoring the analyte ions obtained by the introduction of our standard solutions. Details of the operational settings are listed in Table 1.

**Table 1:** Table 1. Instrumentation and operational settings of ESI-mass spectrometer and Laser.

(1) ESI–MS instrument	QTRAP® system (AB SCIEX, Toronto, Canada)
Operational settings	
Curtain gas	170 kPa psi
Voltage for ion spray	5500 V
Ion source gas 1	76 kPa
Ion source gas 2	0
Declustering potential	100 V
Entrance potential	10 V
Sample uptake rate	6 μL min^−1^
Temperature	393 K
Collision energy	13 V for valine
	24 V for caffeine
	20 V for BBP
Monitored *m*/*z*	Valine: Q1 *m*/*z* 118 Da, Q3 *m*/*z* 72 Da
	Caffeine: Q1 *m*/*z* 195 Da, Q3 *m*/*z* 138 Da
	BBP: Q1 *m*/z 313 Da, Q3 *m*/z 91 Da
(2) Laser ablation system	LAL Fast Scan-355 (JST Corp., Saitama, Japan)
Operational settings	
Wavelength	355 nm
Repetition rate	40 kHz
Fluence	6 J cm^−2^
Raster speed	10 mm s^−1^ (using Galvano scanner)

## RESULTS AND DISCUSSION

### Transmission efficiencies from the sample to the detector

The collection efficiency of the analytes through LAL sampling is very important in terms of evaluating the analytical capability of the technique. Sampling efficiencies of the LAL technique for the three analytes used in this study can be estimated by comparing the ion transmission efficiency of the two sample introduction technique into the mass spectrometer: (i) injection of a standard solution and (ii) injection through LAL sampling. The transmission efficiency from the analyte solution to the ion detector can be defined by calculating the ratio of the number of molecules introduced into the ESI to the number of analyte ions detected by the ion detector. The concentrations of analytes in the standard solutions were all 100 ng mL^−1^, and thus, the calculated numbers of molecules introduced into the ESI were about 5.1×10^10^ molecules s^−1^ for valine, 3.1×10^10^ molecules s^−1^ for caffeine, 1.9×10^10^ molecules s^−1^ for BBP. The measured count rates were 1.8×10^6^ cps (s^−1^) for valine, 1.2×10^6^ cps for caffeine, and 3.5×10^5^ cps for BBP, and thus, the transmission from the analyte solution to the ion detector is 3.5×10^−3^% for valine, 3.9×10^−3^% for valine, 1.8×10^−3^% for BBP, suggesting that the ionisation efficiency of BBP was slightly lower than that for the other two components.

The overall ion yields from solid sample to ion detector including the LAL sampling technique can then be defined by calculating the ratio of the number of molecules collected by the LAL technique to the number of molecules detected by the ion detector. The number of molecules collected through the LAL sampling can be calculated based on concentrations of analytes in the solid materials (5.5 mg mL^−1^ for valine and caffeine, and 10 mg mL^−1^ for BBP) and sampling volume (*i.e.*, 1 mm×1 mm×10 μm): 2.5×10^14^ molecules for valine, 1.5×10^14^ molecules for caffeine, 9.6×10^13^ molecules for BBP. Hence, the depth of the ablation pit sizes was separately measured by taking the difference in the focusing heights for the sample surface and the bottom of the ablation areas using a digital microscope (VHX-8000, Keyence Corp., Osaka, Japan). Possible uncertainties in the depth analysis were about <1 μm when a high-magnification objective lens (×2500, VHX-E2500, Keyence Corp., Osaka, Japan) was employed. The resulting sampling depth can change among materials having various densities, hardness, color, or melting points. To minimise the variations in the sampling depth, the laser ablation with a lower energy fluence (J/cm^2^) can be effective. The major problem associated with laser ablation with a lower fluence is that the sampling volume (and weight) per single laser shot can become low. To overcome this, a high-repetition rate laser (30 kHz) was employed in this study.

In this study, overall ion yields from sample to ion detector are defined based on two assumptions: (a) all sample particles were completely released from the solid materials, and (b) the analytes were extracted into the solvents without any breakdown or decomposition. The LAL sampling technique has been applied for elemental analysis.^18,19)^ Okabayashi and co-workers reported that elements can be extracted and analysed with minimum elemental fractionations (*i.e.*, the tendency of preferential ablation occurring is low).^18)^ Elements have a higher evaporation temperature than organic compounds, and since the elements can be sampled with a low level of elemental fractionation, this led us to conclude that most of the organic compounds could be released from the solid materials. Thus, the sampled weight can be estimated from the sampled volume. However, great care must be placed on the laser-induced breakdown or the decomposition of the organic compounds through laser ablation. Through LAL sampling, both aerosol formation and the dissolution of organic/inorganic components into the solvent proceed at a same time, hence, some of the organic compounds may break down or be decomposed by laser-induced heating during LAL sampling.

Regarding on the extraction and detection of insoluble compounds (*i.e.*, BBP), it is generally thought that BBP is not extracted by water through LAL sampling. It should be noted that the photocurable resin used in this study is not soluble in methanol after the resin is formed, suggesting that the addition of methanol does not cause the release of the BBP irrespective of whether methanol is used instead of water in the LAL ring, nor through the dissolution of the laser-induced sample particles. Rather, BBP is thought to be deposited onto the surface of the laser-induced sample particles during LAL sampling. Hence, BBP can be extracted and analysed by the ESI-MS when methanol is added. The overall ion yields defined in this study can reflect both loss/gains through LAL sampling and sample preparation for the ESI.

Volume of analyte solutions were 200 μL (100 μL of water in the LAL ring plus 100 μL of a 1 : 1 MeOH : H_2_O solution for dilution). Since the sample up-take rate of the ESI system is 6 μL/min, the total time duration for the signal would be about 2,000 s. Based on the measured count rates for analytes and time duration for the signals (*i.e.*, 2,000 s), one can determine the total numbers of molecules detected by the mass spectrometer: 2.8×10^9^ molecules for valine, 1.3×10^10^ molecules for caffeine, and 6.4×10^8^ molecules for BBP. The calculated transmission efficiencies from solid materials to the ion detector for each analyte were 1.1×10^−3^% for valine, 8.7×10^−3^% for caffeine, and 6.7×10^−4^% for BBP. The collection efficiencies of the analytes through LAL sampling can be estimated from the ratio of the transmission efficiencies: 31% for valine, 45% for caffeine, and 37% for BBP, suggesting that more than 30% of the molecules could be recovered through the LAL sampling procedure. We considered the possibility that the recovery of the analytes could change as a function of their solubility in water. The lack in large differences in the collection efficiency for three analytes suggests that the solubility of the analytes did not greatly affect the collection efficiency through LAL sampling. This is in contrast to the LESA technique where solubility is the key parameter that affects recovery.

With the LAL technique, the average size of the laser-induced sample particles ranges from 50 to 100 nm,^18)^ and thus, the presence of the particles within the collected solution will not cause blockage in the nebuliser. An important issue that needs to be considered, however, is the adsorption of the analyte compounds onto the surface of particles. As described in an earlier section, in ESI, only molecules that are dissolved in solvents can be ionised, and therefore, analyte molecules trapped within the particles cannot be ionised, and hence will not be detected. However, the contribution of analytes that are deposited on the particle surface is limited because no significant differences were found in the measured recovery between water-soluble and water-insoluble compounds (*i.e.*, BBP). This lack of difference in recovery suggests that the overall recovery found in this study (*e.g.*, 30–40%) reflects the percentages of ionised or components that are dissolved in water from the solid samples, and thus, the other 60–70% of the organic compounds may have remained within the laser-induced sample particles.

### Repeatabilities of the measurements

Prior to LAL sampling, the sample surfaces were successively cleaned with deionised water and ethanol. In this study, the front ferrule composed of PTFE with the size of 6.35 mm (1/4 inch) (internal volume of the front ferrule is about 200 μL) was used as the sampling ring. The front ferrule was placed on the targeted area of the sample surface. After the addition of 0.1 mL of deionised water to the front ferrule, laser ablation was conducted from a 1 mm×1 mm area through fast scanning using the Galvanometric scanner.^27,28)^ Instrumentation and laser operational conditions are listed in Table 1. The resulting sample solution was collected by a micropipette, and 100 μL of a mixture containing 1 : 1 MeOH : H_2_O and 0.1% HCOOH was then added. To investigate the repeatabilities of the measurements, LAL sampling was conducted five times from separate areas (1×1 mm^2^).

**Figure 3:**
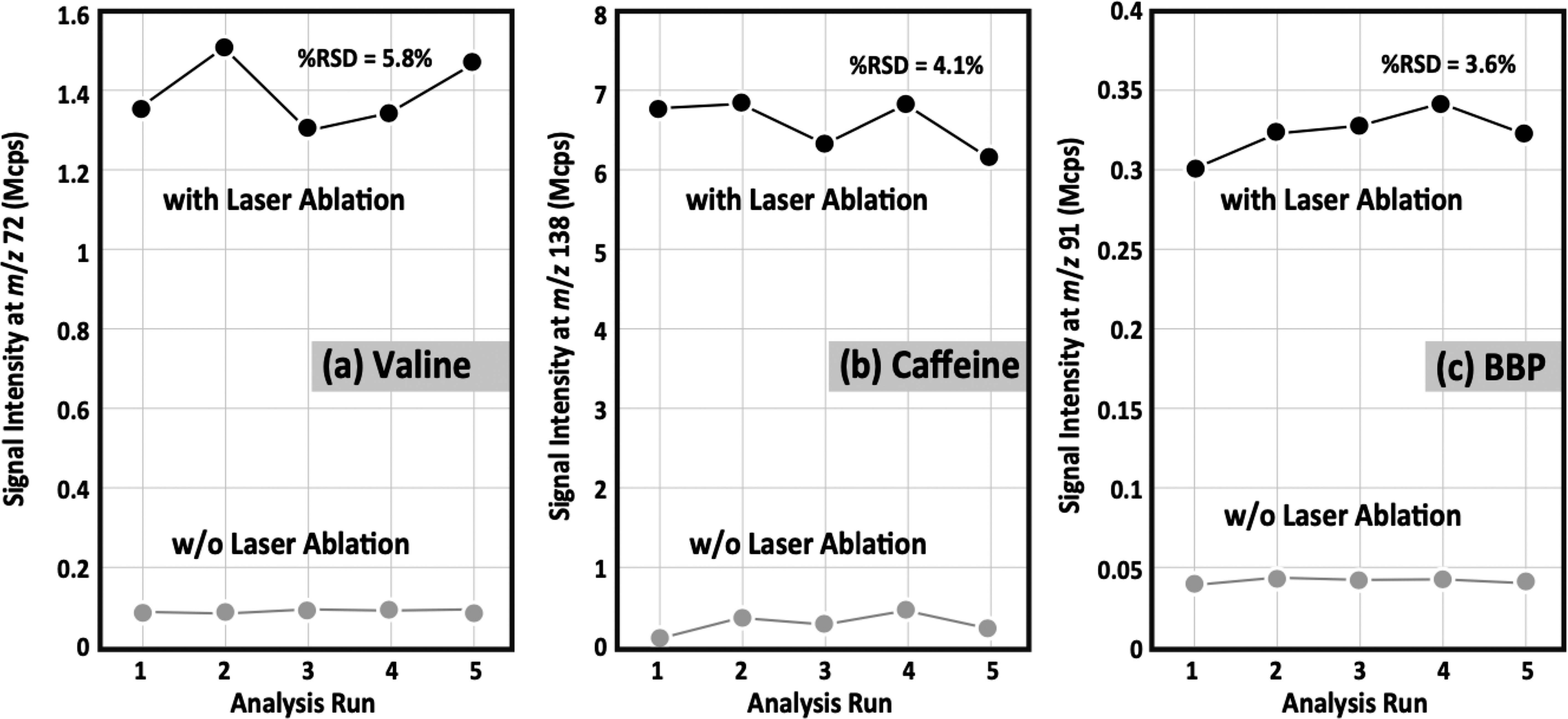
Fig. 3. Comparison of signal intensities for (a) valine, (b) caffeine, and (c) benzyl butyl phthalate (BBP) obtained with laser ablation (black circles) and without laser ablation (grey circles). Analysis was repeated five times to demonstrate analytical repeatability. Note that the BBP is a water-insoluble substance.

The obtained signal intensities for valine, caffeine, and BBP are shown in Fig. 3. The signal intensities obtained without laser ablation are also given in Fig. 3. To obtain reliable blank values, the time duration for introducing water into the LAL ring was adjusted to the same duration as that used for LAL sampling. This is particularly important for water-soluble analytes (*i.e.*, valine and caffeine).

The resulting signal intensities were 1.4 Mcps for valine, 6.5 Mcps for caffeine, and 320 kcps for BBP. These values were significantly greater than those for the blank measurements, demonstrating that the organic components present in the resin standard can be collected by the LAL sampling procedure. The signal intensities for valine and caffeine were significantly greater than that of BBP. The measured difference in the signal intensity values can be attributed to the solubility in water, and the lower signal intensity for BBP and reflects a lower ionisation efficiency in ESI, mainly originating from lower concentration of “free” molecules within the solvent. Despite the obvious lower signal intensities for BBP, the LAL sampling technique can be used for the detection of insoluble components. This is the advantage of the LAL sampling technique over LESA.

The analytical repeatability, as estimated through five repeated sampling from separate sampling areas was 5.8% for valine, 4.1% for caffeine, and 3.6% for BBP. Since BBP has a low solubility in water, this may be the factor for affecting the analytical repeatability of the method. However, this is not the case for the LAL sampling because the repeatabilities of the measurements for BBP were similar to the values for valine and caffeine. The stability in the signal intensity profiles obtained through the solution injection-ESI technique was about 2%, which is significantly better than those for the LAL-ESI technique. The cause of lowering the analytical repeatability can be attributed either to changes in the ionisation efficiency of the analytes or to heterogeneity of the standard materials prepared in this study. It should be noted that the sample preparation procedure for the standard solid materials of BBP was different from those for valine and caffeine. The standard materials for valine and caffeine were prepared through the mixing of solid portions of three components (analyte plus CaCO_3_ and TiO_2_ powder), whereas the standard solid material for BBP was prepared by mixing liquid BBP and CaCO_3_–TiO_2_ powder. Despite the different procedures, the analytical repeatabilities for the three materials did not vary significantly, suggesting that the analytical repeatability is largely dominated by the ionisation process within ESI. To reduce the time-dependent variation in ion yield, the injection of large-sized sample particles needs to be avoided, since this can cause the lowering the plasma temperature of the electrospray plume. At this moment, unfortunately, we do not have any further evidence to support this explanation, so this issue remains as a possibility.

## CONCLUSION

Coupling of the LAL technique and ESI-MS has the potential for being a promising analytical method for the analysis of organic compounds present in solid materials. Through the repeated LAL sampling from in-house solid materials, several findings were made.

1. The overall efficiencies of transmitting solid materials to ion detectors, including LAL sampling, were determined to be 1.1×10^−3^% for valine, 8.7×10^−3^% for caffeine and 6.7×10^−4^% for benzyl butyl phthalate (BBP).
2. Recovery for the LAL sampling for the three analytes were 31% for valine, 45% for caffeine and 37% for BBP. It should be noted that the recovery for the water-insoluble compounds (*i.e.*, BBP) did not vary significantly from those for soluble compounds (valine and caffeine), suggesting that the LAL technique could be used for the analysis of water insoluble compounds.
3. The repeatability of measurements, as estimated by five repeated sampling from separated sampling areas were 5.8% for valine, 4.1% for caffeine, and 3.6% for BBP. The analytical repeatability was governed either by the heterogeneity of the standard materials prepared in this study, or by time-dependent changes in ion yields due to the possible injection of large-sized sample particles into the ESI-mass spectrometer.

The data obtained here clearly demonstrate that the LAL technique has the potential for use as a sampling method for both the soluble and insoluble molecules. LAL has the advantage of being rapid and can be used at atmospheric pressure, without the need for complex and time-consuming sample preparation procedures. Moreover, the collected samples can be stored for lengthy periods of time for use in future analyses.

## Conflicts of Interest

There are no conflicts to declare.

## Notes

Mass Spectrom (Tokyo) 2023; 12(1): A0121
